# An Updated Collection of Sequence Barcoded Temperature-Sensitive Alleles of Yeast Essential Genes

**DOI:** 10.1534/g3.115.019174

**Published:** 2015-07-14

**Authors:** Megan Kofoed, Karissa L. Milbury, Jennifer H. Chiang, Sunita Sinha, Shay Ben-Aroya, Guri Giaever, Corey Nislow, Philip Hieter, Peter C. Stirling

**Affiliations:** * Michael Smith Laboratories, University of British Columbia, Vancouver V6T 1Z4, Canada; ‡ Faculty of Pharmaceutical Sciences, University of British Columbia, Vancouver V6T 1Z4, Canada; ** Department of Medical Genetics, University of British Columbia, Vancouver V6T 1Z4, Canada; † Terry Fox Laboratory, BC Cancer Research Centre, Vancouver, Canada; § Faculty of Life Sciences Bar-Ilan University, Ramat-Gan, Israel

**Keywords:** essential genes, temperature-sensitive alleles, genomics, *Saccharomyces cerevisiae*

## Abstract

Systematic analyses of essential gene function using mutant collections in *Saccharomyces cerevisiae* have been conducted using collections of heterozygous diploids, promoter shut-off alleles, through alleles with destabilized mRNA, destabilized protein, or bearing mutations that lead to a temperature-sensitive (ts) phenotype. We previously described a method for construction of barcoded ts alleles in a systematic fashion. Here we report the completion of this collection of alleles covering 600 essential yeast genes. This resource covers a larger gene repertoire than previous collections and provides a complementary set of strains suitable for single gene and genomic analyses. We use deep sequencing to characterize the amino acid changes leading to the ts phenotype in half of the alleles. We also use high-throughput approaches to describe the relative ts behavior of the alleles. Finally, we demonstrate the experimental usefulness of the collection in a high-content, functional genomic screen for ts alleles that increase spontaneous P-body formation. By increasing the number of alleles and improving the annotation, this ts collection will serve as a community resource for probing new aspects of biology for essential yeast genes.

Analysis of essential genes in model organisms has lagged behind that of nonessential genes because of the inherent difficulty in conducting genetic experiments on strains lacking a protein required for cell growth. Systematic strategies to partially reduce the function of essential genes are available in various forms in the yeast *S. cerevisiae* [*e.g.*, as heterozygous diploids, Decreased Abundance by mRNA Perturbation (DAmP), Doxycycline-repressible, temperature-sensitive (ts), and ts Degron] (Kanemaki *et al.* 2003; Baetz *et al.* 2004; Giaever *et al.* 2004; Mnaimneh *et al.* 2004; Ben-Aroya *et al.* 2008; Breslow *et al.* 2008; Li *et al.* 2011) and exist in other organisms using, for example, systematic RNAi or CRISPR interference-based approaches (Kamath *et al.* 2003; Gilbert *et al.* 2014). The components of certain biological processes or structures are encoded almost exclusively by essential genes (*e.g.*, the proteasome, the kinetochore), and therefore analysis of nonessential genes fails to interrogate important areas of biology. Moreover, essential genes are more conserved across evolution than nonessential genes (Hughes 2002; Jordan *et al.* 2002), making the study of essential genes critical in model organisms where many research goals depend on learning by orthology to other organisms, especially humans.

Temperature-sensitive (ts) alleles provide unique and complementary advantages compared to other technologies for studying essential genes. The ts alleles usually confer the ability to inactivate a gene both conditionally and completely (*i.e*., to push cells to lethality or work under semipermissive conditions) without changing media, adding chemicals, or drastically altering the transcriptional context of the gene of interest. For this reason, recent years have seen the creation of ts allele collections covering large segments of yeast essential genes (Ben-Aroya *et al.* 2008; Li *et al.* 2011). We previously reported the “diploid-shuffle” method for ts allele construction and created a collection of 250 ts mutants (Ben-Aroya *et al.* 2008; Ben-Aroya *et al.* 2010b). These mutants have been used for dozens of research studies including systematic functional screens for phenotypes as diverse as chromosome instability, chemical genetic screens, and killer toxin K28 sensitivity (Ben-Aroya *et al.* 2008; Carroll *et al.* 2009; Stirling *et al.* 2011; van Pel *et al.* 2013).

Here we report the final version of our “diploid-shuffle”–based ts allele collection that now contains inactivating mutant alleles for 600 essential yeast genes. This collection contains mutants in >50% of yeast essential genes and represents essential biological processes uniformly. The collection is available in an arrayed format and genetic background suitable for high-throughput strain construction. We also provide a comprehensive overview of the different alleles currently available for the essential yeast genome. We hope that by describing this updated collection, providing more information about the allele composition and behavior, and demonstrating the utility of the collection for screening, it will continue to serve the community as a resource for biological discovery.

## Materials and Methods

### Yeast growth and strain construction

Yeasts were grown according to standard protocols on the media and temperatures indicated. Double mutants for validation of genetic interactions between *lsm1*Δ and splicing factors were created by tetrad dissection. For spot dilution assays strains were normalized to the same OD600, then serially diluted 10-fold, and transferred to test media using a 48-pin metal manifold (Dan Kar Corporation).

### Sequencing of ts alleles

Freshly grown 1536 density arrays grown on YPD at 25° were scraped using sterile water. Genomic DNA was extracted using two rounds of phenol-chloroform extraction and ethanol precipitation essentially as described (Hoffman and Winston 1987). PCR was performed using primers OPH7402 and OPH1810 that are common to the sequences flanking the alleles in SB221 (OPH7402 anneals in a sense orientation within the KanMX gene: 5′-GGTCAGACTAAACTGGCTGAC-3′; OPH1810 anneals downstream of the URA3 start codon in an antisense orientation: 5′-GGATGAGTAGCAGCACGTTCC-3′). The amplicons were subjected to Charge Switch PCR cleanup (Life Technologies) and converted to a sequencing library using the Nextera XT library preparation kit (Illumina). This library was sequenced on a single MiSeq lane (Illumina), generating 150-bp paired-end reads. MiSeq reporter was used to align reads to the Saccharomyces cerevisiae reference genome (www.yeastgenome.org) using the resequencing pipeline, which uses the Burrows-Wheeler Algorithm (BWA) to create alignments. Sequence data are deposited (http://www.ncbi.nlm.nih.gov/bioproject/) with bioproject accession PRJNA283554. The resulting bam file was then annotated with SnpEff (Cingolani *et al.* 2012). Variants were manually confirmed by examining aligned sequence reads in the Integrated Genomics Viewer (Robinson *et al.* 2011; Thorvaldsdottir *et al.* 2013). Variant functional impacts were predicted using the SiFT Blink tool (http://sift.jcvi.org/www/SIFT_BLink_submit.html) (Kumar *et al.* 2009).

### Protein structure analysis

PDB files 1W1W for Mcd1 and 1I3Q for Rpb8 were downloaded from the protein data bank (www.rcsb.org) (Cramer *et al.* 2001; Haering *et al.* 2004). PDBs were oriented and annotated in DeepView (www.expasy.org/spdbv) before producing figure images using POV-ray (www.povray.org/).

### Barcode microarray

Ten freshly grown 1536 density cell arrays were pooled by flooding the plates with YPD and scraping into sterile YPD. Genomic DNA was isolated as above. For competitive growth experiments aliquots of the pooled collection were outgrown at 25° for 210 min before being diluted and shifted to the assay temperature for 20 generations in YPD. Genomic DNA was extracted as above and fed into the established barcode PCR and microarray hybridization protocol (Pierce *et al.* 2007; Hoon *et al.* 2008). Because the essential gene collection contained only 1200 possible barcodes, arrays were cohybridized with barcodes derived from the nonessential homozygous deletion pool to reduce nonspecific binding. Analysis of 3′ and 5′ barcodes was conducted independently because of the potential loss of 5′ barcodes discussed in the text. Values from triplicate experiments at each temperature were averaged for comparison.

### P-body formation screen

A MATα strain carrying *LSM1*-GFP::*HIS3MX* from the yeast GFP collection (Huh *et al.* 2003) was transformed with *Eco*RI digested p4339 to switch the His3MX marker to NatMX (Tong *et al.* 2001). Because the ts collection is a query array, it has all of the necessary markers to conduct high-throughput strain construction when mated to any appropriately marker MATα haploid. Construction of a Lsm1-GFP::NatMX + ts allele output array was performed at 384 density by mating on YPD, selection of diploids on SC-URA-NH_4_SO_4_ + glutamate + nourseothricin (Werner BioAgents), sporulation on enriched sporulation media, and haploid selection on SC-URA-HIS-ARG- NH_4_SO_4_ + glutamate + nourseothricin using the protocols described in (Baryshnikova *et al.* 2010) and a Singer Rotor (www.singerinstruments.com/). The haploid output array was selected by serially pinning on haploid selection media and dearraying to 96 density.

The strains from one 96 density array plate were transferred to liquid SC-URA media, grown overnight at 25°, diluted to an OD of ∼0.2 at 25° the following day, and allowed to grow for 4 hr at 25° to obtain log phase cells. Batches of 12 wells were shifted to a fresh 96-well plate at 37° for 2 hr. 12-well Teflon masked slides were pretreated with 50 μg/mL concanavalin A (Sigma) to bind treated cells prior to imaging the medial focal plane at 100× on a Zeiss Axioscop (Carroll *et al.* 2009; Stirling *et al.* 2012). At least two fields of view were captured in this primary screen and candidate strains with >15% of cells showing Lsm1-GFP foci were retested, capturing a greater number of images to allow quantification of the frequency of P-body formation.

### Data availability

All strains and materials are available upon request. Table S1 contains the strain identifications and plate map for the allele collection. Table S2 shows enriched gene ontology terms from the collection. Table S3 summarizes sequence changes identified by sequencing of ts alleles. Sequence data for this experiment is deposited with bioproject accession PRJNA283554. Table S4 contains averaged barcode microarray data for each condition listed in the paper. Table S5 shows the percent of cells with P-bodies at high temperature for those mutants with significantly more P-bodies than wildtype. Table S6 provides a summary of all mutant allele collections for analysis of essential genes in yeast.

## Results and Discussion

### Assembly and characterization of a diploid shuffle ts collection

**Figure 1:**
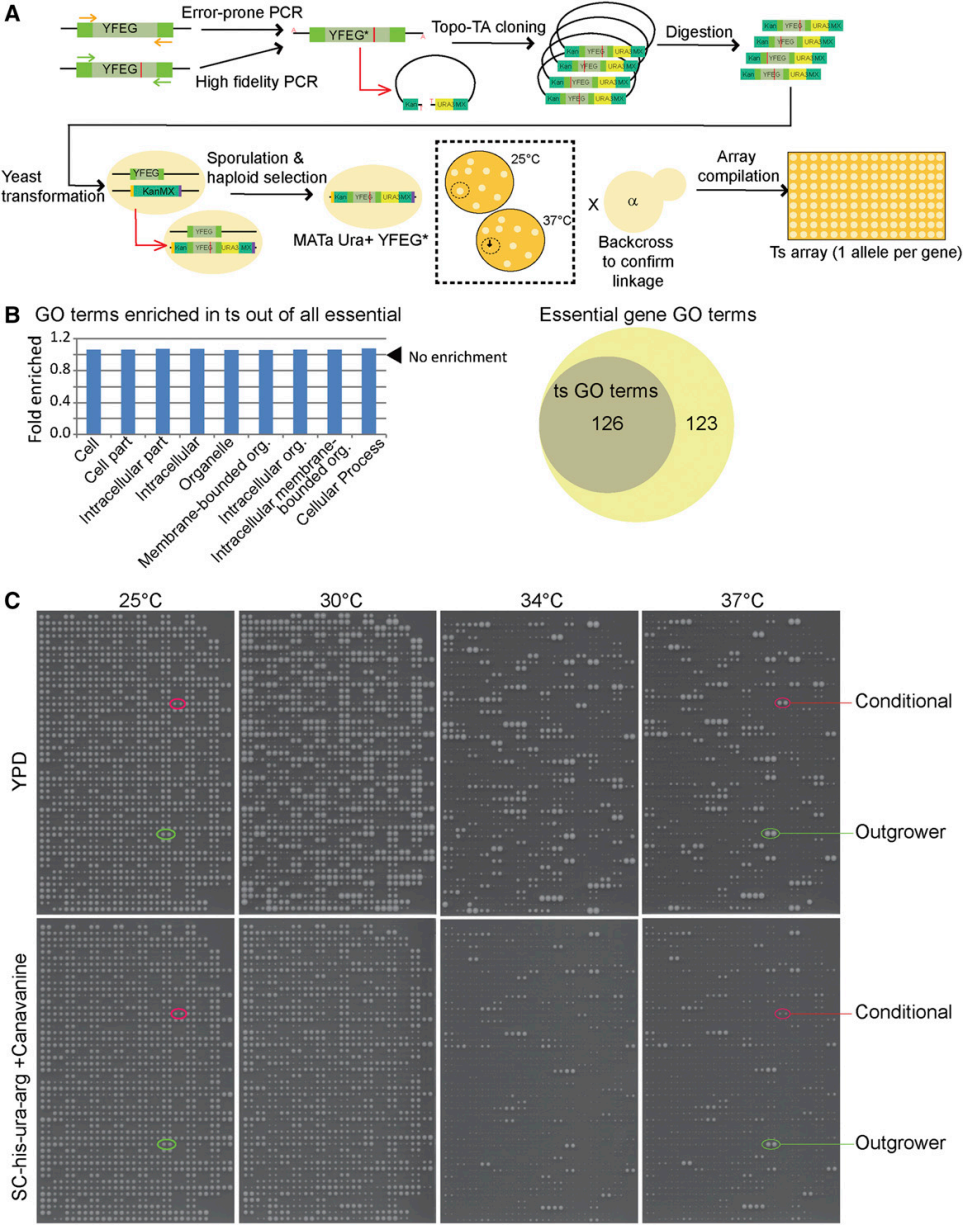
Generation and characterization of a diploid shuffle ts allele array. (A) Schematic of the diploid shuffle method. *De novo* ts alleles are generated by error-prone PCR of YFEG (any essential gene), whereas preexisting ts alleles are amplified by high-fidelity PCR. The resulting product is ligated into plasmid SB221 by Topo-TA cloning. A library of mutant genes is excised by restriction digest and targeted to KanMX in the corresponding YFEG heterozygous diploid deletion strain. Sporulation and haploid selection create a library of mutant strains from which ts alleles are selected by replica plating to high temperatures (dotted box). Candidates are outcrossed to confirm linkage of the ts phenotype to the *URA3* marker and positives are compiled in an array. (B) Enriched gene ontology (GO) terms in the ts allele collection out of all essential genes. Left panel: Only generic high-level terms are enriched in the ts collection using all essential genes as a background set. Right panel: GO terms retrieved from the whole genome by the ts alleles overlap completely with those retrieved by all essential genes. (C) Allele behavior in high-density colony arrays. 1536 density arrays with each allele represented twice were pinned at the indicated temperatures onto rich media (upper panel) or magic media (lower panel). Highlighted are examples of conditional ts behavior (*i.e.*, growth on YPD but not magic media) and of alleles with normal fitness at low temperatures that exploited the lack of competition at high temperatures (*i.e.*, outgrowers).

To expand our previously published resource we targeted genes for which no previous ts allele has been described and performed diploid shuffle using error-prone PCR and screening for temperature-sensitivity following haploid selection (Figure 1A) (Ben-Aroya *et al.* 2008). To supplement this resource with an even larger set of ts alleles in a common strain background, we also directly transferred alleles from a published ts allele resource using high-fidelity PCR into the diploid shuffle plasmid SB221 for integration (Li *et al.* 2011). In general, two to eight ts clones were stored and backcrossed to a donor strain to test: (1) the ts phenotype segregates in a Mendelian manner indicative of a single mutated gene; (2) the ts phenotype is linked to *URA3* and therefore cosegregates with the mutagenized PCR product; and (3) the mutagenized PCR product is integrated at the correct locus, rending the cells G418 sensitive. When a new or transferred haploid strain bearing a ts allele was confirmed in this manner, single ts alleles were retained in a mutant array for high-throughput manipulation and analysis (Supporting Information, Table S1). In most cases during creation of the *de novo* ts alleles multiple candidates were frozen, sometimes up to 12 independent alleles. Although only a single allele was validated by linkage and included in the arrayed collection, the other candidates are available on request for specific genes if an investigator is interested in an allelic series.

In total we have assembled a collection containing ts alleles in 600 unique essential genes, representing >50% of all essential genes in yeast. Importantly, this allele collection is not unduly biased to any particular biological functions. Comparison of Gene Ontology (GO) terms associated with all essential genes and those genes in the ts collection show no enrichment of specific terms (Table S2). The reverse experiment, looking for GO term enrichment among essential genes not found in the ts collection, retrieved no enriched terms at all. The enrichment of a few generic terms such as “Cellular Process” or “Organelle” within the ts collection is likely because genes in the collection are typically better annotated than the average of all essential genes (Figure 1B). Searching for enriched GO terms genome-wide using the 600 ts allele genes, or all essential genes from the yeast knockout project as queries yields largely similar results: all enriched GO terms retrieved using the 600 ts genes are found in the essential gene set (Figure 1B, Table S2). Overall, this indicates that screens of this ts collection should be relatively comprehensive with respect to pathways because the alleles represent all annotated biological pathways among essential genes.

To assess the behavior of the ts alleles in the context of the mutant array, we pinned the collections on rich and synthetic media across different permissive, semi-permissive, and nonpermissive temperatures. Most alleles showed a dramatic loss of viability between 30**°** and 34**°** and almost all strains showed defects in growth at 37**°** (Figure 1C). Some strains were able to capitalize on the lack of competition from neighboring colonies and actually grew larger on the array plate. We also noted that many alleles grew better on rich media (YPD) than on synthetic media, which is not surprising given that ts behavior of the alleles was originally selected on synthetic haploid selection media, not YPD (Figure 1C, conditional). When we examined some of the colonies able to grow at 37**°** by spot dilution assays, we found that some alleles are only ts on synthetic media (Figure S1). These results are an important reminder of the environmental context-dependence of some phenotypes. Moreover, spot assays showed us that many alleles retain their ts behavior in direct tests, although they are able to grow better than their competitors on arrays. A minority of alleles appear to develop suppressors after selection at high temperature, which is always a caveat when working with ts alleles.

### Pooled sequencing of the ts alleles

Knowing the amino acid sequence of a ts allele allows the application of structure–function analyses to interpret the phenotypes of a given allele in light of protein structure information. We used flanking primers common to each allele to amplify the alleles from pooled genomic DNA *en masse* for deep sequencing. This strategy captured full-length, high-quality sequence for 300 ts alleles (Table S3); the remaining half were not included because of either low sequence quality or no sequence reads. In the latter case, some of the alleles may not have amplified because of biases in the PCR reaction, but many appear to have lost the unselected 5′ region intervening between the duplicated gene promoters, which would prevent annealing of the common 5′ primer (see below section on barcode microarrays).

**Figure 2:**
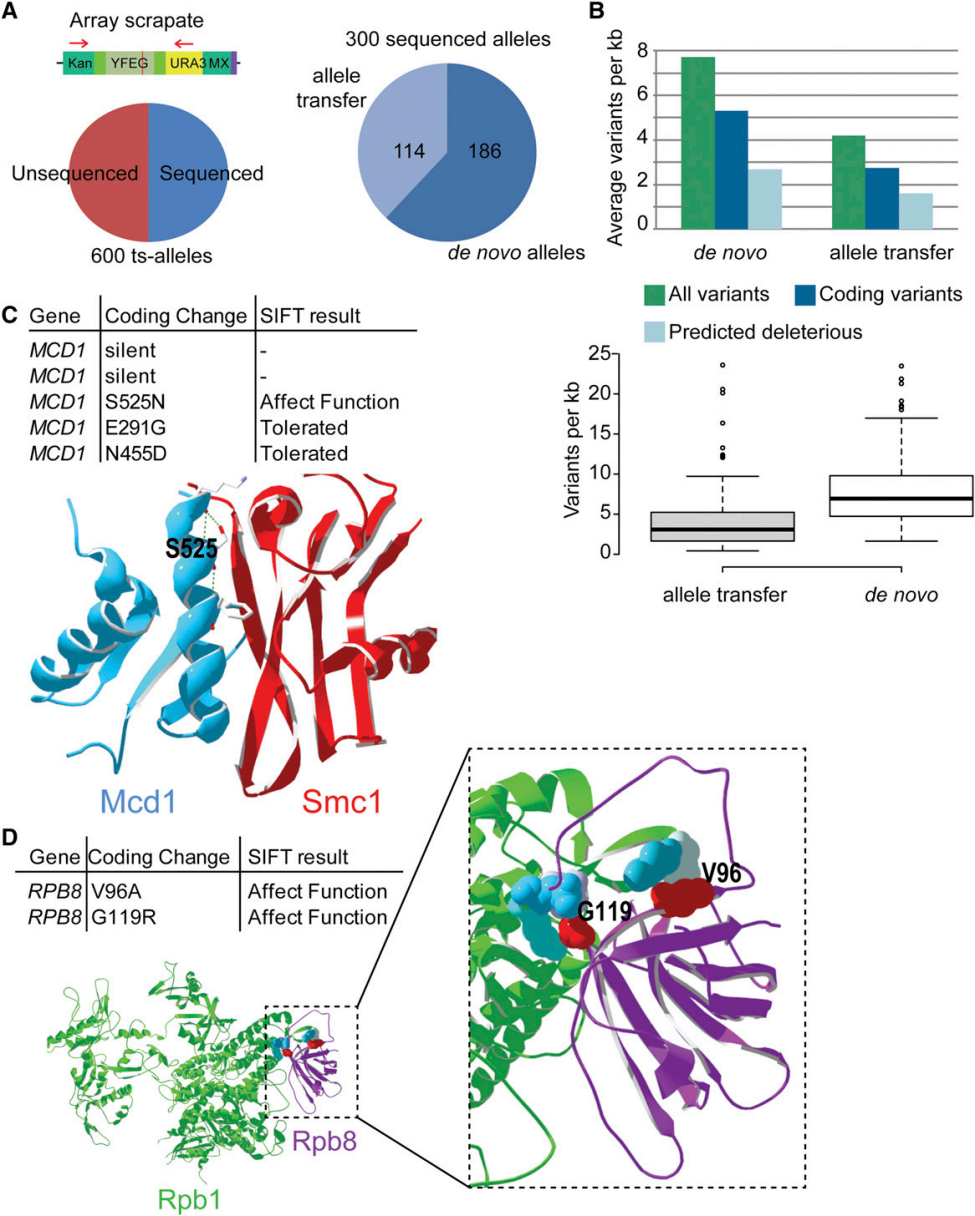
High-throughput ts allele sequencing. (A) Sequencing strategy and overall results. Genomic DNA from the array scrapate was subjected to PCR with flanking primers common to each allele (red arrows), followed by Illumina sequencing. High-quality sequence for 300 of 600 alleles was obtained in this manner, including 186 *de novo* constructed alleles and 114 alleles from Li *et al.* (2011). (B) Variant density in each allele population. Upper panel: Average variants per kilobase. Lower panel: Distribution of variant densities across alleles shown in box plots. For box plots, center line shows the median, box limits are the 25^th^ and 75^th^ percentiles, whiskers extend 1.5× the interquartile range, and dots represent outliers. (C and D) Examples of variant functional reports from Table S3 with corresponding structural investigation. (C) A single mutation predicted to affect function in Mcd1 (Blue) lays at the interfacial α-helix contacting Smc1 (red) (Haering *et al.* 2004). (D) Two mutations predicted to affect function in the RNA polymerase II subunit. Rpb8 (purple) lays its interface with Rpb1 (green).

Of the 300 alleles sequenced, on average we saw nearly six variants per 1 kb, of which four variants introduced protein coding changes (Figure 2A). In general, alleles selected by diploid-shuffle had more variants (7.7/kb) that those transferred from the compiled community ts collection (4.2/kb), probably reflecting the different mutagenesis strategies used (Figure 2B). Although the modal allele has three coding variants and the median allele bears five such variants, extreme cases exist; seven alleles had more than 15 coding variants and *fol1*-ts bears the most, with 30 coding variants across its 2475-bp length (Table S3). As expected, one-third of the mutations were synonymous, usually reflecting silent transition mutations at the wobble position. It is important to note, however, that each allele bore at least one coding variant, and we predict that the silent/synonymous mutations rarely if ever contribute to the ts phenotype.

Having protein sequence information functionalizes the collection to interpretation of the ts phenotype in important ways. Most importantly is that structure–function studies on many of the mutations are now possible and will enable dissection of crucial regions of each protein. To aid identification of the amino acid changes that cause loss-of-function, computational approaches such as SIFT (Sorting Intolerant From Tolerant) or Mutation Assessor are available. These approaches primarily use evolutionary conservation as a means to predict protein variant impact (Kumar *et al.* 2009). Applying SIFT to all missense mutations in the set of 300 ts alleles predicted that more than half of variants are likely tolerated. After SIFT the modal number of variants predicted to affect protein function was reduced to one, whereas 236 of 300 alleles are predicted to have three or fewer variants that affect protein function (Figure 2B). Indeed, 105 alleles are predicted to be affected by only a single coding change (Table S3). In some cases these predictions coincide with known structural features. For example, one of three mutations in *mcd1*-ts is predicted to affect its function and the predicted deleterious variant, S525N, lies at the protein–protein interface with Smc1 (Figure 2C) (Haering *et al.* 2004). Serine 525 is involved in hydrogen bonding to stabilize the alpha helix in which it resides, and three of five rotamers of the S525N substitution create predicted steric clashes. Similarly, the *rpb8*-ts allele bears G119R and V96A substitutions, both of which are predicted to affect function, and both appear precisely at the Rpb8-Rpb1 interface in the crystal structure, suggesting that protein complex formation may be disrupted (Figure 2D) (Cramer *et al.* 2001). Many such structure–function predictions will be possible using this resource and the knowledge of individual labs because at least 216 of the sequenced alleles have structural homologs in the Protein Data Bank (www.yeastgenome.org). Recreation of single SIFT-predicted deleterious alleles may reconstitute the ts phenotype and eventually provide more refined tools (*i.e.*, single point mutants) for the community to analyze loss of essential gene functions.

### Integrity and application of flanking sequence barcodes in the ts collection

**Figure 3:**
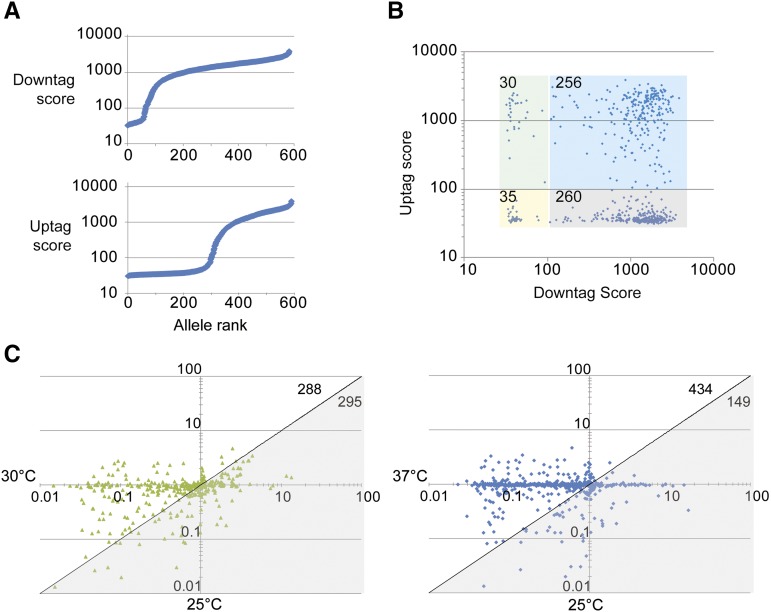
Detection of flanking barcodes in ts alleles by microarray. (A) The raw score of the barcodes detected in a pooled scrapate sorted by intensity for the 3′ downtag (top) and the 5′ uptag (bottom). (B) Comparison of downtag and uptag scores for each allele. 581 alleles had both barcodes represented on the microarray. For our purposes an arbitrary score cut-off of 100 was applied to group the data. Colored boxes indicate the groups chosen and the number of alleles in each group is shown in the upper left. Although most alleles retain a functional downtag, less than half have a functional uptag. (C) Competitive growth analysis of the ts allele collection outgrown at the indicated temperature. The microarray intensity score of each allele under control or experimental conditions is expressed as a ratio. The gray triangle highlights scores lower at 25°, whereas the white area shows higher scores. The numbers of alleles in each group are noted on the upper right. Note the shift of alleles into the white area at 37°. The average of three replicates is used in (C) and all axes are logarithmic scales.

One potentially useful byproduct of the diploid-shuffle method is to link each ts allele to the cognate barcode assigned to the heterozygous deletion mutant corresponding to the gene of interest (Figure 1). However, because the promoter region of each ts allele is duplicated upstream of the 5′ barcode in the genome, the alleles may be integrated in such a way that the 5′ barcode is deleted or subsequently lost due to direct repeat recombination between the duplicated promoter (Figure 1A). The 3′ barcode from the deletion strain being targeted should remain intact due to selection of the *URA3* marker that sits between the direct repeats on the 3′ end of both the allele and the original deletion strain. To measure the integrity of the barcodes and determine their use in pooled growth experiments, we performed barcode microarrays (Giaever *et al.* 2002) of the pooled ts collection either directly after scraping from the array plate or after 20 generations of growth at various temperatures. Analysis of the ts collection scrapate confirmed that the 3′ barcodes (downtags) were more likely to be intact (Figure 3A and Table S4). More than 500 of the 581 alleles with downtags on the array met our arbitrary cutoff of normalized signal intensity 100, whereas less than half of the 5′ barcodes (uptags) met this threshold (Figure 3A). Both tags were present for 256 of the alleles, whereas 260 had only downtags and only 30 scored strongly for uptags without having a detectable downtag (Figure 3B). Comparing the behavior of downtags after 20 generations of outgrowth across temperature showed a temperature-dependant loss of tag intensity. The ratios of tag scores at 25**°** and 30**°** cluster around 1, whereas the ratios of 37**°** to 25**°** are shifted to less than 1 (Figure 3C). Together these data show that most alleles in the collection can be interrogated using their 3′ barcode and open the door to pooled growth analyses of the ts collection under various environmental or stress conditions.

### Identification of essential pathways suppressing P-body formation

P-bodies have emerged as general cellular stress responders that promote RNA sequestration and degradation. Although many types of stress induce P-bodies, one unexpected recent observation is that P-bodies are required for the cellular response to DNA replication stress (Herrero and Moreno 2011; Tkach *et al.* 2012). Moreover, P-body genes appear as hubs in genetic interaction networks of genome maintenance factors, suggesting that their role in genome stability could be important (van Pel *et al.* 2013). As such, we used P-bodies as a test case for a functional genomics analysis using the ts allele collection by conducting a cytological screen for those alleles that increase the presence of P-bodies at a nonpermissive temperature. This visual screen was initiated by introducing an *LSM1-GFP*::*NatMX* construct to the ts collection using high-throughput strain construction (Tong *et al.* 2001). Cells from the ts allele Lsm1-GFP output array were grown in 96-well plates and shifted to 37**°** for 2 hr prior to live cell imaging. After the temperature shift, 15% of WT cells contained P-bodies and this served as our baseline for calling hits in the primary screen. Candidates from the primary screen were retested and quantified as a percentage of cells exhibiting P-bodies in a medial focal plane. Remarkably, after validation, we found that ts alleles in 169 unique genes (28%) significantly increased the frequency of P-bodies in cells, ranging from 28% to 98% of cells showing P-bodies (Table S5).

**Figure 4:**
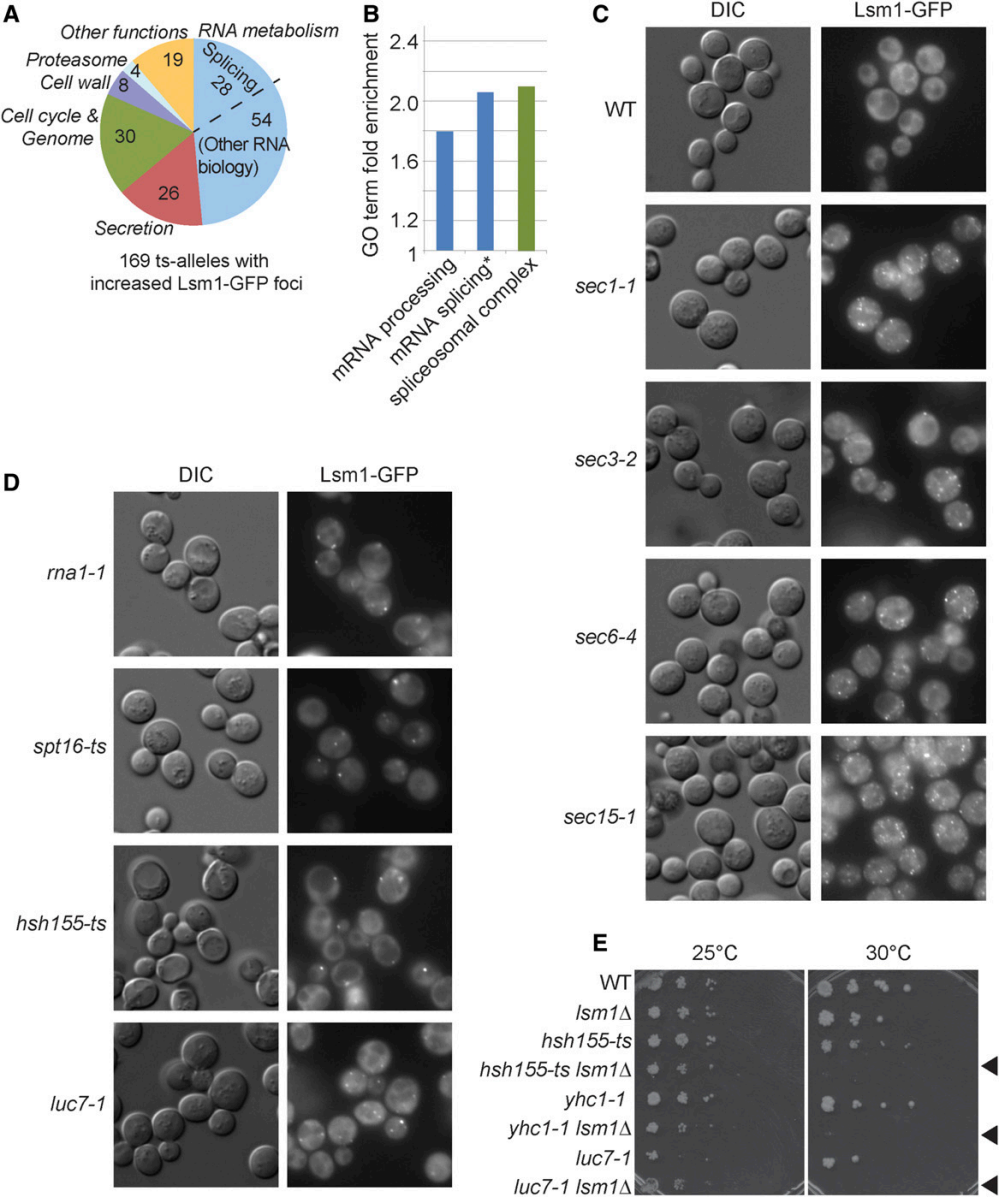
Cytological analysis of P-body formation in each ts allele. (A) Overall functional groupings of 169 genes whose mutation increases spontaneous P-body formation. 82 genes functioning in RNA metabolism were identified (light blue), 28 of which function in splicing. (B) GO term enrichment among P-body forming mutants. Using the alleles tested as a background set, only mRNA processing and splicing-related terms are significantly enriched. *Full name of the term is *mRNA splicing*, *via spliceosome*. (C and D) Examples of the varied morphology of P-body induction phenotypes. (C) Typical P-body induction in secretion mutants where many punctate Lsm1-GFP speckles form. (D) Several different RNA processing mutants where P-bodies are usually present as a single focus or as a few foci in each cell. Images in (C) and (D) were taken after a 2-hr temperature shift from 25° to 37°. (E) Spot dilution assays of splicing mutants identified in the Lsm1-GFP foci screen with or without the *LSM1* gene. Black arrows indicate the double mutant strains are unable to grow at the normally permissive temperature of 30°.

There are several possible reasons why the number of P-body-inducing mutants is so high. One explanation could be that this enrichment is independent of gene function and reflects the fact that some of the alleles encode transcripts with nonsense mutations that are targeted for degradation by P-bodies. However, we do not favor this explanation because only 3 of 23 ts alleles in which stop codons are gained or lost exhibit high levels of P-bodies, arguing against a general role for nonsense transcripts inducing P-bodies in our screen. Alternatively, because the ts alleles impair essential functions when shifted to 37°, indirect stress responses that change the transcriptome and induce P-bodies are very likely (Decker and Parker 2012). Despite the possible indirect effects, several processes emerge as highly prone to induce P-bodies upon disruption. As expected, a large number of the alleles (49%) have known roles in RNA metabolism, considered broadly to include transcription, mRNA processing, splicing, rRNA biogenesis, and histone modification (Figure 4, A and B, Table S5). Another large group of P-body–forming mutants function in secretion. In total, 26 mutants in genes with known secretory functions increased P-body formation. Interestingly, Lsm1-GFP localized to multiple small puncta rather than the small number of bright P-bodies seen in splicing mutants (Figure 4C). This "speckled" Lsm1-GFP phenotype was only found in secretory mutants and is consistent with previous observations that secretory defects led to multiple Dcp2-GFP marked P-bodies and that P-bodies may actually assemble at the endoplasmic reticulum membrane (Kilchert *et al.* 2010).

Among RNA processing mutants, splicing was particularly enriched, with ts alleles in 28 splicing genes inducing P-bodies. In fact, only the spliceosome and RNA processing/splicing-related GO terms appear as significantly enriched when treating the 600 ts alleles screened as a background gene set (Figure 4B). In contrast to secretion mutants, defects in RNA biology genes induced a small number of bright Lsm1-GFP foci with little background as shown for mutants of splicing factors (*HSH155*, *LUC7*), RNA transport (*RNA1*), and transcription (*SPT16*) (Figure 4D).

While Lsm1 and splicing are both involved in RNA processing, unlike Lsm2-8, Lsm1 does not have a direct role in splicing. Nevertheless, degradation of improperly spliced transcripts has been shown to occur in the cytoplasm (Hilleren and Parker 2003). More recently, the spliceosome has also been implicated in nonintronic mRNA degradation through so-called spliceosome-mediated decay, although this may involve primarily nuclear mRNA surveillance mechanisms (Volanakis *et al.* 2013). Given the induction of P-bodies by splicing mutants, we asked whether *LSM1* becomes required for viability in splicing mutants. Strains with mutations in splicing factors *LUC7*, *HSH155*, and *YHC1* revealed synthetic growth defects at semi-permissive temperatures (30°) when lacking *LSM1*, highlighting the need for P-body function in mutants with disrupted splicing (Figure 4E). It is possible that either the parallel action of P-body and spliceosome-mediated decay in RNA turnover or the need to turnover potentially deleterious improperly spliced transcripts in the cytoplasm explain both the P-body induction and genetic interactions of splicing mutants.

### Perspective

Our ability to systematically assess the function of genes, especially in high-throughput experiments, is critical to creating a complete functional model of the cell (Boone 2014; Giaever and Nislow 2014; Rine 2014). The collection of ts alleles described here is amenable to high-throughput genetic screens, chemical screens (van Pel *et al.* 2013), pooled growth experiments, and diverse phenotypic and functional studies, including those for chromosome instability, RNA processing, killer toxin sensitivity, and others (Ben-Aroya *et al.* 2008; Carroll *et al.* 2009; Ben-Aroya *et al.* 2010a; Stirling *et al.* 2011; Albulescu *et al.* 2012; Minaker *et al.* 2013). Therefore, it opens up new prospects for understanding the biology of most essential pathways in budding yeast and ultimately will contribute to the complete functional map of the cell. Multiple powerful resources now exist to study essential genes using various technologies. Table S6 lists the systematic strain collections available; only 18 essential genes lack a mutant allele and 76% of genes have three or more mutant alleles. Further characterization of these collections and the expansion of other mutant resources for studying essential genes will continue to provide new and tractable tools in yeast biology for the community.

## Supplementary Material

Supporting Information
