# Supplementary material for: An Updated Collection of Sequence Barcoded Temperature-Sensitive Alleles of Yeast Essential Genes
Source: G3 (Bethesda). 2015 Jul 14;5(9):1879–87. doi: 10.1534/g3.115.019174 (PMC4555224; doi:10.1534/g3.115.019174)
Supplement: Supporting Information [file supp_5_9_1879__index.html]

An Updated Collection of Sequence Barcoded Temperature-Sensitive Alleles of Yeast Essential Genes — Supporting Information 

# An Updated Collection of Sequence Barcoded Temperature-Sensitive Alleles of Yeast Essential Genes

## Supporting Information for Kofoed *et al.*, 2015

**Files in this Data Supplement:**

- Supporting Information - Figure S1 and Tables S1-S6 (PDF, 1 MB)
- Figure S1 - Spot dilution assays confirming varied ts-allele behavior seen in high density arrays. (PDF, 1 MB)
- Table S1 - Plate map of ts-alleles. (.xls, 96 KB)
- Table S2 - GO term analysis of the ts-collection against all essential genes. (.xls, 49 KB)
- Table S3 - Sequence analysis for 300 ts-alleles. (.xls, 603 KB)
- Table S4 - Barcode score summary. (.xls, 440 KB)
- Table S5 - Results of Lsm1-GFP P-body formation screen. (.xls, 35 KB)
- Table S6 - Summary of essential gene mutant collections. (.xls, 187 KB)
