# Supplementary material for: An Updated Collection of Sequence Barcoded Temperature-Sensitive Alleles of Yeast Essential Genes
Source: G3 (Bethesda). 2015 Jul 14;5(9):1879–87. doi: 10.1534/g3.115.019174 (PMC4555224; doi:10.1534/g3.115.019174)
Supplement: Supporting Information [file supp_g3.115.019174_019174SI.pdf]

G3: Genes | Genomes | Genetics

An updated collection of sequence barcoded temperature-sensitive alleles of yeast essential genes

Megan Kofoed[1], Karissa L. Milbury[2], Jennifer H. Chiang[3], Sunita Sinha[3], Shay Ben-Aroya[4], Guri Giaever[3] Corey Nislow[3], Philip Hieter[1,5], Peter C. Stirling[2,5]

[1]Michael Smith Laboratories, University of British Columbia, Vancouver, Canada
[2]Terry Fox Laboratory, BC Cancer Research Centre, Vancouver, Canada
[3]Faculty of Pharmaceutical Sciences, University of British Columbia, Vancouver, Canada
[4]Faculty of Life Sciences Bar-Ilan University, Ramat-Gan, Israel
[5]Department of Medical Genetics, University of British Columbia, Vancouver, Canada

Correspondence to Philip Hieter (Hieter@msl.ubc.ca) or Peter Stirling (pstirling@bccrc.ca)

DOI: 10.1534/g3.115.019174

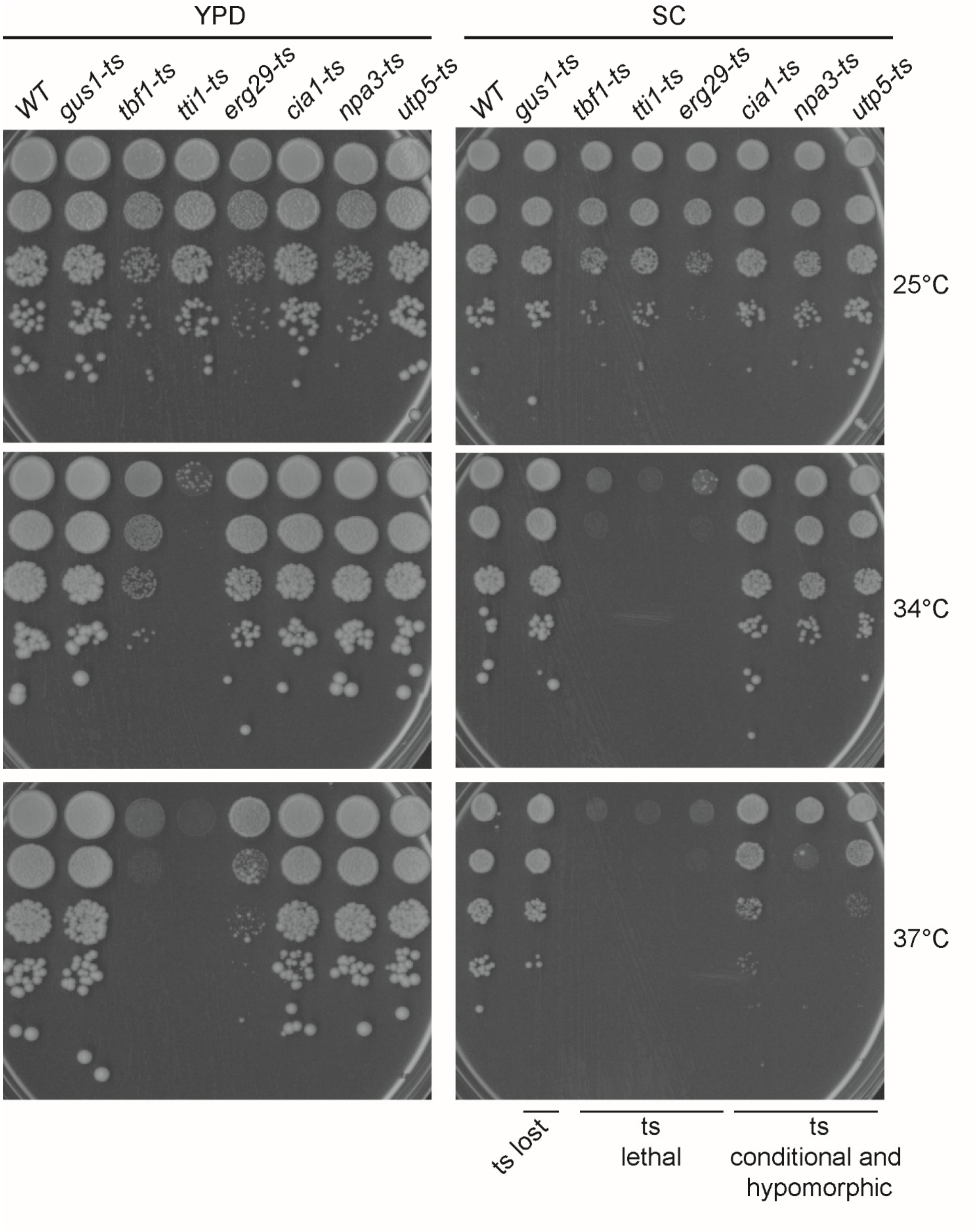

Figure S1 Spot dilution assays confirming varied ts-allele behavior seen in high density arrays. Ten-fold serial dilution spot assays of the indicated strains were performed across temperature on the indicated media. Strains were picked from the compiled ts-collection array and tested for growth. Stable lethal (*tbf1*-ts, *tti1*-ts, *erg29*-ts) and hypomorphic (*cia1*-ts, *npa3*-ts, *utp5*-ts) phenotypes are evident. Revertants and suppressors are also possible when working with ts-cell populations from high-density arrays; *gus1*-ts was originally isolated as slow growing at 34°C, but the isolated clone is able to grow robustly at 37°C.

Tables S1-S6
Available for download as Excel files at www.g3journal.org/lookup/suppl/doi:10.1534/g3.115.019174/-/DC1

Table S1. Plate map of ts-alleles

Table S2. GO term analysis of the ts-collection against all essential genes

Table S3. Sequence analysis for 300 ts-alleles

Table S4. Barcode score summary

Table S5. Results of Lsm1-GFP P-body formation screen

Table S6. Summary of essential gene mutant collections
