# Supplementary material for: An Updated Collection of Sequence Barcoded Temperature-Sensitive Alleles of Yeast Essential Genes
Source: G3 (Bethesda). 2015 Jul 14;5(9):1879–87. doi: 10.1534/g3.115.019174 (PMC4555224; doi:10.1534/g3.115.019174)
Supplement: Supporting Information [file supp_g3.115.019174_FigureS1.pdf]

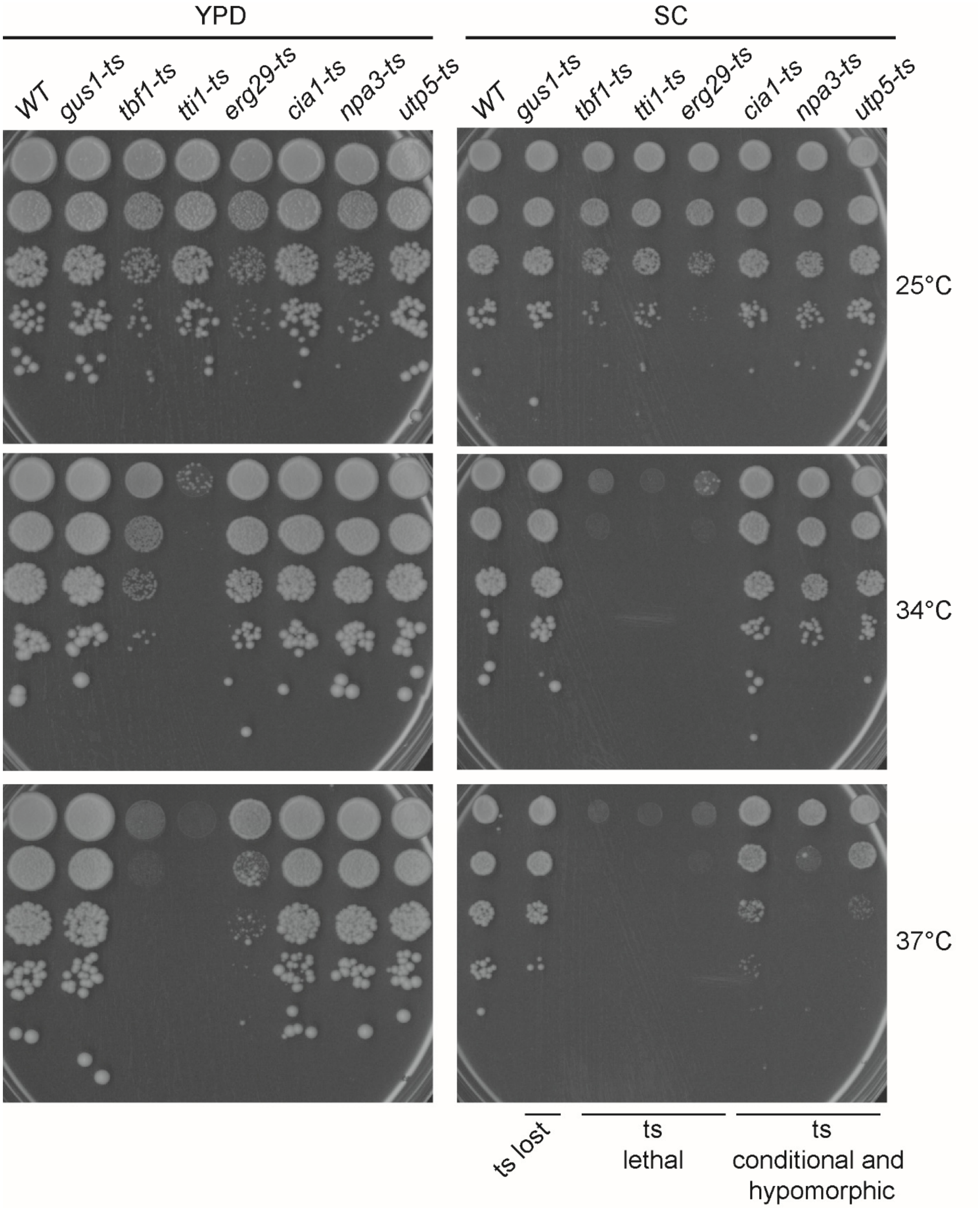

Figure S1 Spot dilution assays confirming varied ts-allele behavior seen in high density arrays. Ten-fold serial dilution spot assays of the indicated strains were performed across temperature on the indicated media. Strains were picked from the compiled ts-collection array and tested for growth. Stable lethal (*tbf1*-ts, *tti1*-ts, *erg29*-ts) and hypomorphic (*cia1*-ts, *npa3*-ts, *utp5*-ts) phenotypes are evident. Revertants and suppressors are also possible when working with ts-cell populations from high-density arrays; *gus1*-ts was originally isolated as slow growing at 34°C, but the isolated clone is able to grow robustly at 37°C.
